# Case report: A simple and reliable approach for progressive internal distraction of the sternum for Jeune syndrome (asphyxiating thoracic dystrophy): preliminary experience and literature review of surgical techniques

**DOI:** 10.3389/fped.2023.1253383

**Published:** 2023-09-26

**Authors:** Alessandro Inserra, Angelo Zarfati, Valerio Pardi, Arianna Bertocchini, Antonella Accinni, Ivan Pietro Aloi, Cristina Martucci, Simone Frediani

**Affiliations:** ^1^ General and Thoracic Pediatric Surgery Unit, Bambino Gesù Children’s Hospital, IRCCS, Rome, Italy; ^2^ University of “Tor Vergata”, Rome, Italy

**Keywords:** Jeune syndrome, sternal distractor, thoracic dystrophy, thoracic insufficiency syndrome (TIS), children

## Abstract

**Background:**

Described for the first time in 1954, Jeune syndrome (JS), often called asphyxiating thoracic dystrophy, is a congenital musculoskeletal disease characterized by short ribs, a narrow thorax, and small limbs. In this study, we analyzed and presented our preliminary experience with a device for progressive internal distraction of the sternum (PIDS) in patients with symptomatic JS. In addition, we reviewed the contemporary English literature on existing surgical techniques for treating children with congenital JS.

**Material and methods:**

A retrospective analysis of pediatric patients (<18 years old) treated for symptomatic JS at our tertiary center between 2017 and 2023 was performed.

**Results:**

We presented two patients with JS who underwent surgery using an internal sternal distractor, a Zurich II Micro Zurich Modular Distractor, placed at the corpus of the sternum among the divided halves.

**Conclusions:**

We obtained promising results regarding the safety and effectiveness of this less-invasive device for PIDS in patients with symptomatic JS. Further studies on long-term outcomes are needed to validate these findings.

## Introduction

Described for the first time in 1954, Jeune syndrome (JS), often called asphyxiating thoracic dystrophy, is a congenital musculoskeletal disease marked by short ribs, a narrow thorax, and small limbs (1). Patients may also have non-skeletal-associated anomalies, such as impaired renal function, liver fibrosis, pancreatic cysts, and retinal anomalies (2, 3). The syndrome is exceedingly rare, with an estimated incidence of 1 per 100,000/130,000 live births (2, 3), and has a genetic basis. Moreover, JS is considered an autosomal recessive ciliopathy with a variable degree of clinical expression.

Genes encoding intraflagellar transport proteins have been implicated in etiopathology. Mutations in other genes have not been excluded and may be implicated, but their roles in the pathogenesis remain to be identified. Moreover, the disease is part of the heterogeneous spectrum of thoracic insufficiency syndrome (TIS), which includes any disorder resulting in an inability of the chest to support normal pulmonary development, function, and growth (4, 5). According to the classification elaborated by Campbell and Smith, JS mostly manifests as a chest deformity caused by lateral maldevelopment and is classified as Type IIIb among the TIS (4). The presentation and symptoms may vary from slightly symptomatic to lethal in early infancy (3). Notably, the respiratory distress is caused by the anomalous morphology of the thorax, predisposing to a progressive restrictive respiratory insufficiency (3, 6). Highly symptomatic patients experience severe lung hypoplasia, pulmonary hypertension, and restrictive respiratory insufficiency. Importantly, the respiratory status is evolving and progressive, with a rapid deterioration of lung function, which typically ends in demise during the first few months (2). A considerable risk of respiratory deterioration during follow-up exists even for children with mild or intermediate forms at diagnosis, who typically acquire recurrent lung infections (3).

There is no consensus or guideline on the ideal timing or approach for surgical treatment, owing to the rarity of the syndrome. Several techniques have been proposed for the surgical management of patients with symptomatic JS, aiming to increase pulmonary expansion. Some require a median sternotomy (7–16), while others are based on thoracotomy and thoracic expansion [lateral thoracic expansion (2, 17–21), vertical thoracic expansion (22–26), and others based on sternal/rib elevation (27–29)].

Most median sternotomy methods rely on prosthesis (either synthetic or autologous) interposition (7–9, 11, 15, 16, 30). These approaches, which widen the sternal halves to enlarge the chest, may result in thoracic growth and expansion. However, even if good outcomes are achieved, they may be transient. Indeed, to achieve general somatic growth of a child, progressive distraction and/or staged procedures may be necessary to allow for progressive chest development. The use of an external distractor has occasionally been reported to overcome this issue (11–13). However, because these tools are taken from surgery on other body parts, they are often disproportionate for the small chests of newborns, infants, toddlers, and children with JS. To limit invasiveness, we developed a new minimally invasive approach using an extremely small device (Zurich II Micro Modular Distractor, KLS Martin Group, Germany) for progressive internal distraction of the sternum (PIDS) for JS.

In this study, we analyzed and presented our preliminary experience with a minimally invasive device for PIDS in patients with symptomatic JS. We also reviewed the contemporary English literature on existing surgical techniques for treating children with congenital JS.

## Methods

We present two cases of pediatric patients (<18 years old) treated for a symptomatic JS at our tertiary center (General and Thoracic Paediatric Surgery Unit, Bambino Gesù Children's Hospital, IRCCS, Rome, Italy) between 2017 and 2023. Clinical, imaging, and surgical data were retrieved from medical charts and radiological systems for patients included in the study. Follow-up outcomes were also assessed. Surgical complications were classified according to the Clavien–Dindo classification (31).

### Operative technique

#### Device placement

**Figure 1:**
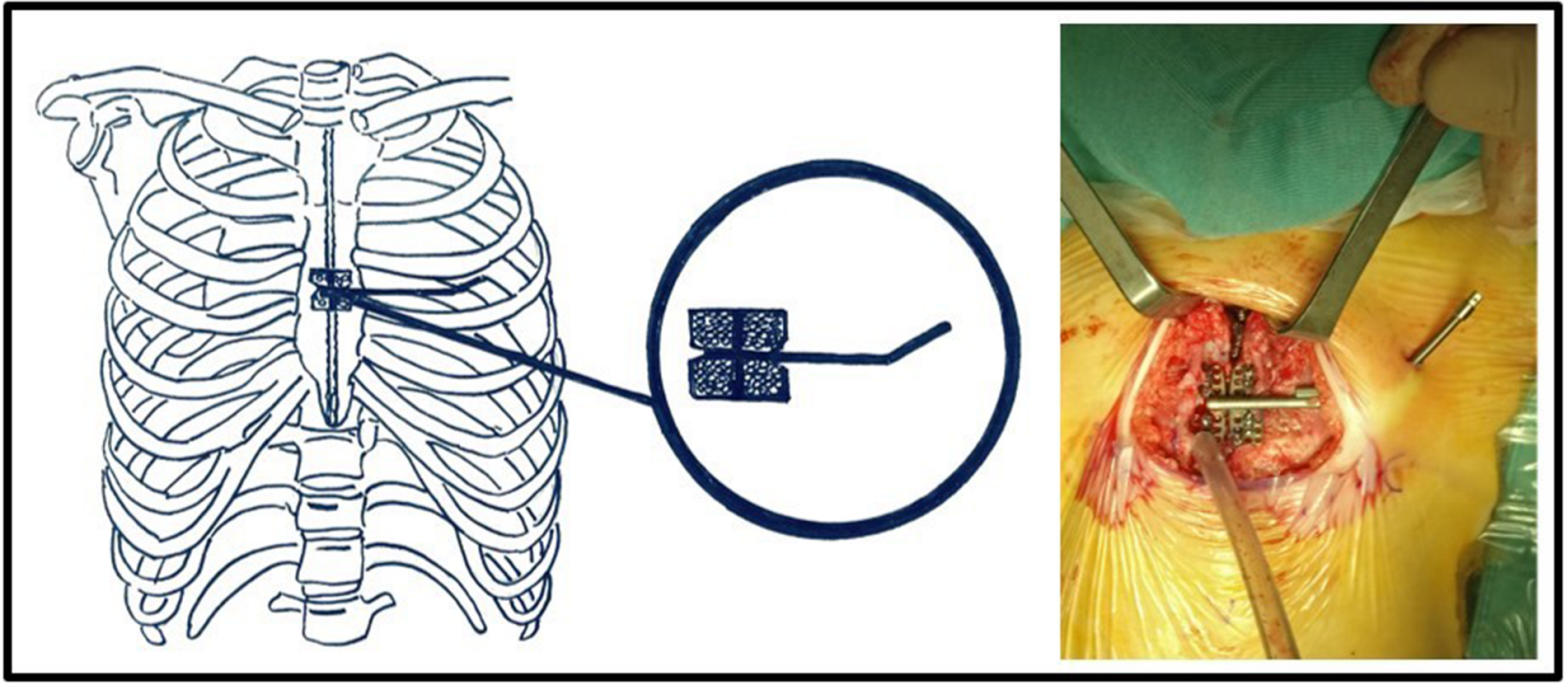
The internal sternal distractor (Zurich II Micro Zurich Modular Distractor - KLS Martin Group, Germany).

The patient was positioned supine with the head hyperextended, the chest elevated to 30°, and a roll placed under the shoulders. The skin was incised at the midline from the sternal notch to the tip of the xiphoid process. Cautery was used to incise and separate the subcutaneous tissue, divide the pectoralis muscle in the midline, and score the sternum at the periosteum. A space was bluntly created at the top of the sternal notch, behind the sternal manubrium, and inferiorly until the tip of the xiphoid process. A sternal saw was used to divide the sternum. Bleeding from the sternal edges was controlled using osseous wax. The internal sternal distractor used at our institution was the Zurich II Micro Zurich Modular Distractor (KLS Martin Group, Germany) (Figure 1). The device was placed on the corpus of the sternum between the divided halves. The lateral end of the distractor was exited from a different incision along the midclavicular line. Furthermore, the device was fixed to the sternal halves with eight screws on each side. The sternal halves were positioned 2 mm apart intraoperatively. A silicone membrane was placed over the device to prevent decubitus movement. The incision was closed in layers.

#### Postoperative care

The patient was hospitalized in the intensive care unit (ICU). The sternal distraction protocol was initiated on postoperative day 1 if the patient was clinically stable. The external arm of the device was rotated clockwise in one turn (360° = 1 mm) daily. Distraction continued until the intended spread of the sternum was reached. The desired spread depended on the age of the patient , respiratory status, and the severity of the defect. Moreover, patients were transferred from the intensive care unit to the ward when they were clinically stable.

#### Distractor removal

The same incision was made in the first procedure. The device was then isolated carefully. The screws were removed. The distractor was then freed from the sternum and removed. Good hemostasis is crucial. The residual cavity was washed with povidone-iodine solution, and the incision was closed in layers over a Penrose drain and left in place until productive.

#### Literature review

We conducted a non-systematic review of the contemporary English literature (from PubMed) to examine the reported surgical techniques for treating congenital JS in pediatric patients. A classification system was developed based on the surgical approach and technique principles outlined in Table 1. The operative techniques were categorized into three main groups: Group I consisted of sternal spreading through median sternotomy, Group II involved thoracic expansion via thoracotomy, and Group III encompassed alternative techniques that raised the sternum or ribs without sternotomy or thoracotomy. These main groups were further divided to provide a more precise classification. Within Group I, we created two subgroups: Subgroup Ia included techniques utilizing a prosthesis, while Subgroup Ib involved using progressive distraction devices, known as distractors. Group II was subdivided into two subgroups based on the direction of expansion: IIa denoted lateral thoracic expansion, and IIb indicated vertical thoracic expansion. This classification also respects the chronological order of the proposal: group I [Todd et al. (1986) (15)], group II [Davis et al. (1995) (17)], and group III [Fette and Rokitansky (2005) (27)].

**Table 1:** Classification of surgical techniques and literature review.

Groups		Subgroups			First proposal		Case report/Series (cases)
I	Sternal spread via median sternotomy	Ia	Prosthesis	Methyl methacrylate	Todd et al. (1986) (15)		• Todd et al. (1986 ) (1) (15) • Takada et al. (1994) (1) (16) • Sharoni et al. (1998) (1) (7)
				Homologous bone	Aronson et al. (1999) (8)		• Aronson et al. (1999) (1) (8)
		Ib	Progressive distraction devices	External	Park et al. (2015) (11)		• Park et al. (2015) (1)(11) • Imai et al. (2016) (1) (12) • Temel and Akgül (2021) (1) (13)
				Internal	Custom-made	Kaddoura et al. (2001) (9)	• Kaddoura et al. (2001) (1) (9)
					Leibinger craniofacial	Conroy et al. (2010) (10)	• Conroy et al. (2010) (1) (10)
					Micro-modular	Present series	• Present series (2)
II	Thoracic expansion via thoracotomy	IIa	Lateral		Davis et al. (1995) (17)		• Davis et al. (1995) (1) (17) • Davis et al. (2001) (10) (18) • Andrade et al. (2011) (1) (20) • Muthialu et al. (2014) (9) (21) • Lena et al. (2022) (7) (2)
		IIb	Vertical		Campbell et al. (2004) (22)		• Campbell et al. (2004) (27 thoracic insufficiency syndrome: number of JS non-specified) (22) • Waldhausen et al. (2007) (2) (23) • Lacher and Dietz (2011) (1) (24) • Betz et al. (2014) (19) (25) • O’Brien et al. (2015) (24) (26)
III	Sternal/ribs elevation (no sternotomy/thoracotomy)	Four steps thoracoplasty (titanium plates, implants, and stabilizers)			Fette and Rokitansky (2005) (27)		• Fette and Rokitansky (2005) (1) (27)
		Nuss procedure			Kikuchi et al. (2010) (28)		• Kikuchi et al. (2010) (2) (28)
		Fixation with a double-angled mandible locking plate			Drebov et al. (2017) (29)		• Drebov et al. (2017) (1) (29)

## Results

### Case presentation

#### Case 1

A 2-year-old girl patient with a prior diagnosis of JS was referred to our tertiary center for assessment. The child was born at 40 weeks, weighing 4,100 g, following a difficult delivery due to abnormal progression. At birth, the neonate exhibited macrocephaly, anomalies affecting the thorax and limbs, and an extra finger. Diagnostic and genetic tests confirmed JS. The child had a history of recurrent bronchitis episodes, necessitating multiple hospitalizations. On two occasions, viral infections (rhinovirus and respiratory syncytial virus) led to acute respiratory failure. In the current case, the child responded well to high-flow nasal cannula (HFNC) therapy and supportive care. Given the heightened risk of respiratory deterioration, she was referred to our tertiary center at the age of 2 for potential surgical evaluation.

**Figure 2:**
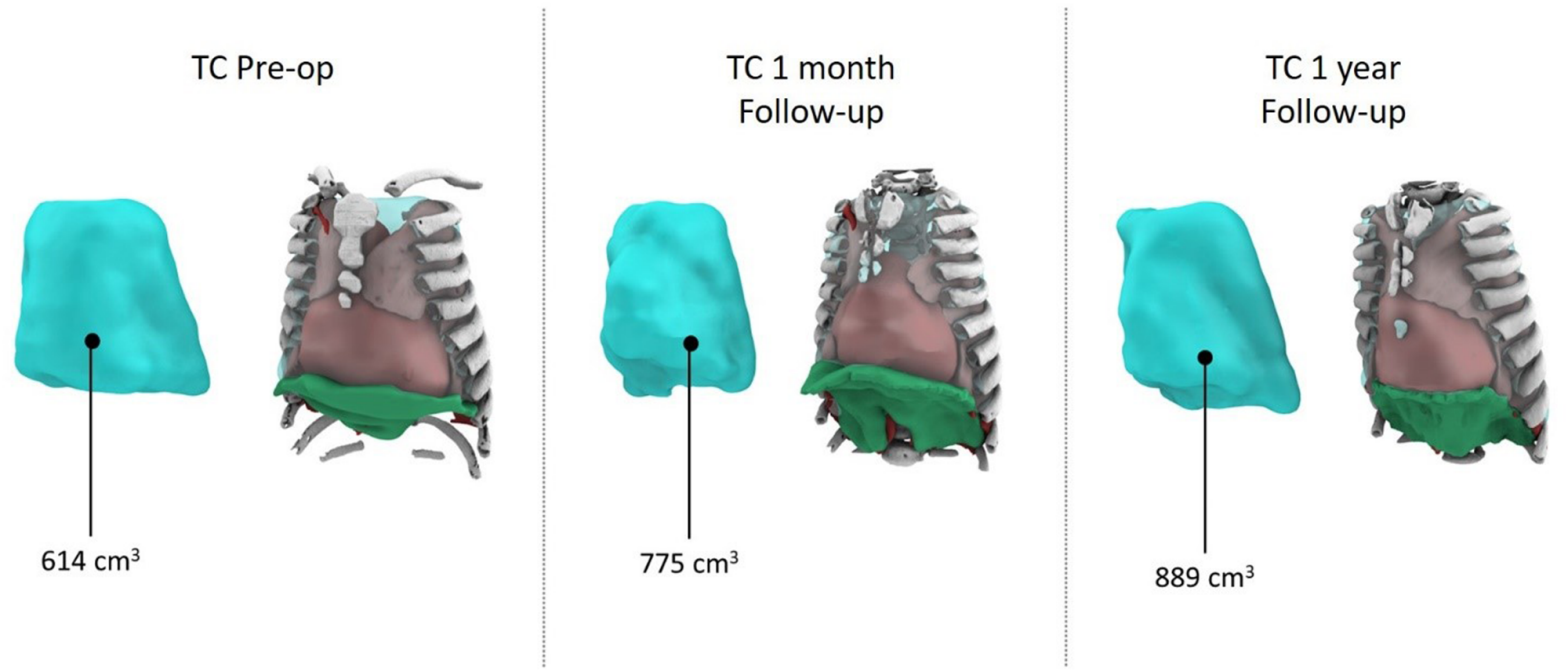
Thorax volume pre and post surgery.

A comprehensive cardiorespiratory study indicated prolonged hypopnea episodes with notable hypercapnia and desaturation. Non-invasive 24-h respiratory support was initiated. After a multidisciplinary discussion considering the child's respiratory status, surgery was deemed favorable due to rapid clinical decline. The surgical procedure, involving device placement using the aforementioned technique, lasted 63 min and was without complications. The patient was observed in the ICU postoperatively, where progressive distraction began on the first day. An episode of *Pseudomonas aeruginosa* respiratory infection complicated the ICU stay but was managed effectively with antibiotics and supportive care. Following extubation, 24-h non-invasive ventilation was introduced. The child transitioned to the ward on day 14 and eventually underwent daily dilations until a 4-cm spread was achieved, following the procedure outlined in the international literature. A chest CT after dilation demonstrated substantial chest volume enlargement and improvement in previously affected lung areas (Figure 2).

**Figure 3:**
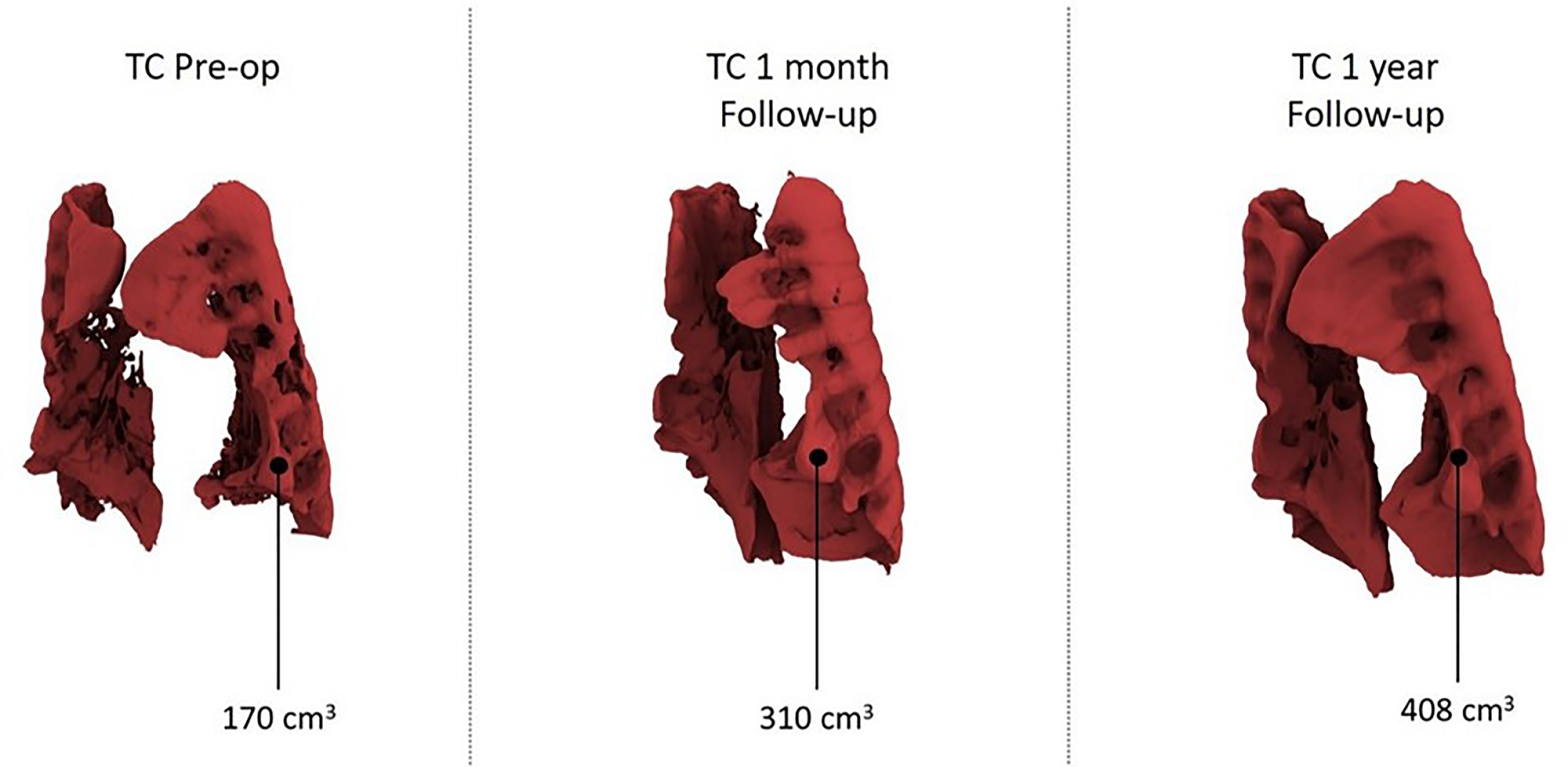
Total lung volume pre and post surgery.

According to CT volumetry, the patient exhibited an 87% increase in pulmonary volume (Figure 3). A subsequent nocturnal cardiorespiratory study under non-invasive ventilation revealed notable improvements in cardiorespiratory values without apnea/hypopnea, although some hypoventilation and suboptimal exchange persisted. The device was removed after exactly 60 days using the same technique. The procedure took 73 min without perioperative complications, and the patient was discharged on day 19, tolerating non-invasive ventilation exclusively during nighttime.

**Figure 4:**
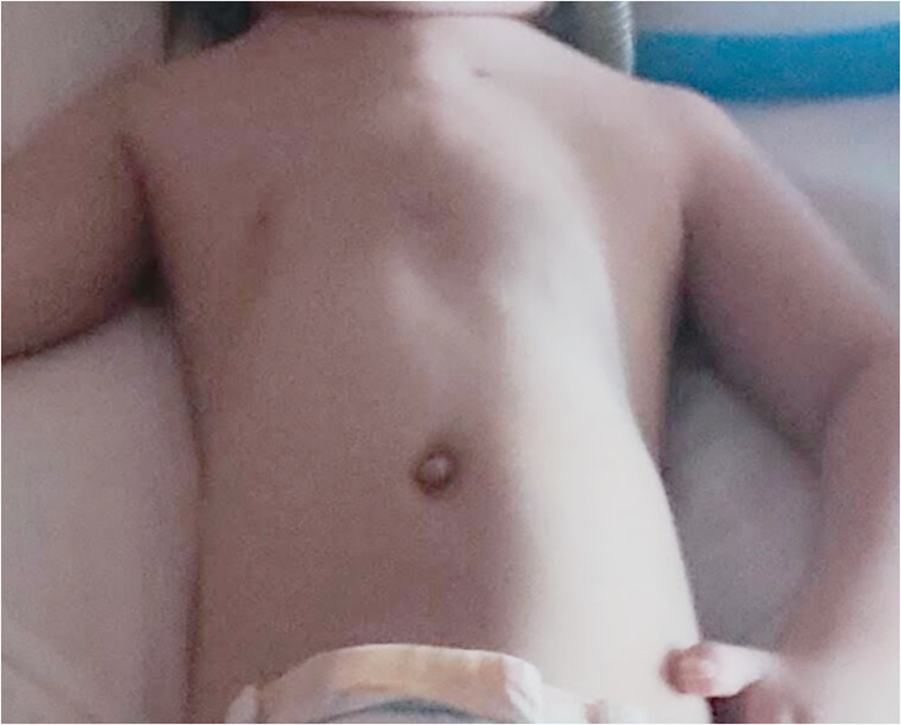
The thorax of the baby 2 years after surgery.

Regular follow-ups included CT scans postdilation and radiological evaluations. The patient, now 5.5 years old, exhibited stable respiratory status during the 3-year follow-up period (Figure 4). Two reactivation episodes featuring hypoxia and hypercapnia necessitated hospitalization, both effectively managed by adjusted, non-invasive ventilation. No additional surgical interventions were required.

#### Case 2

A 6-month-old girl infant affected by JS was transferred to our tertiary center in serious clinical condition to our intensive care unit. The patient was born at term (3,230 g) via physiological delivery. Six hours after birth, the respiratory status deteriorated, and the newborn was intubated. Physical examination revealed significant skeletal and limb anomalies. A complete diagnostic workup, including imaging and genetic examinations, was performed. The clinical and radiological findings were highly suggestive of JS. Genetic testing confirmed a diagnosis of JS. The newborn required 6 days of intubation and an additional 9 days of oxygen therapy. Her respiratory status progressively improved, and she was discharged without oxygen support.

She experienced a first moderate episode of acute respiratory insufficiency at 2 months of age. Instead, at 6 months, she experienced a more severe episode requiring ICU hospitalization. The infant was initially unstable and had a critical clinical condition. She was transferred to our tertiary center for non-invasive ventilation and eventual surgical treatment when possible. The case was discussed in a multidisciplinary manner, and an indication for surgery was established. After 10 days, the infant was considered sufficiently stable for the procedure. The placement was performed according to the aforementioned technique. The distractor procedure required 63 min. The patient did not develop any immediate complications. The patient was hospitalized in the ICU for the first postoperative period. Non-invasive ventilation was delivered initially for 24 h. Successful passage to nocturnal non-invasive ventilation and initiation of the distraction protocol were possible on postoperative day 7. During progressive distraction, the infant experienced progressive amelioration of her respiratory status during the daytime and later at night. The device was kept in place for 74 days. The distractor was removed according to the abovementioned technique and took 42 min without perioperative complications. The infant was discharged on postoperative day 14. At discharge, the child tolerated the non-invasive ventilation well only for night hours.

The child was regularly followed up for 6 years after surgery. The patient was re-evaluated periodically in a multidisciplinary manner. At the final follow-up visit, the patient's respiratory status was stable. The patient did not require further surgical treatment, and an adequate contour of the anterior chest wall was maintained.

### Literature review

A literature review is presented in Table 1. We divided the techniques into three main groups based on the new classification system defined above.

Group I contains the first reported technique for correction (15). Subgroup Ia includes techniques using a prosthesis, and subgroup Ib uses devices for progressive distraction, also known as distractors. The use of a methyl methacrylate prosthesis was the first successful technique reported for the correction of JS (15) and was among the most reported in the past (7, 16). The use of a homologous bone for the same scope has also been described (8). These approaches, which widen the sternal halves to enlarge the chest, may result in thoracic growth and expansion. However, even if good outcomes are achieved, they may be transient. Indeed, to achieve a child's general somatic growth, progressive distraction and/or staged procedures may be necessary to allow for progressive chest development. Recently, some techniques for progressive distraction have been proposed (Ib) (9–13). Distraction may be realized through an external (11–13), or internal device (9, 10). Due to the lack of specific instruments for internal sternal distraction, surgeons used custom-made devices (modified Bailey Rib Approximator) (9) or devices originally developed for different uses (10), as in the present series. However, the disproportionate size of these adapted devices increases the invasiveness and morbidity associated with this approach. Therefore, we developed a new, less-invasive approach using an extremely small device (Zurich II Micro Modular Distractor, KLS Martin Group, Germany) adapted from mandibular surgery. Our approach may be considered a modification and evolution of the approach of Conroy et al.(10). These authors first reported using a Leibinger modular internal distractor (MID; Stryker Leibinger, Germany), which has been used in craniofacial disorders.

Group II was more homogeneous and included the most reported cases. This group may be subdivided according to the direction of the expansion into two subgroups: IIa with lateral thoracic expansion, first proposed in 1995 by Davis et al. (17), and IIb with vertical thoracic expansion, described in 2004 by Campbell et al. (22). Both the lateral and vertical thoracic expansions are the most reported techniques for JS correction (2, 17, 18, 20–26).

Group III was the least heterogeneous group of classifications and included sternal or rib elevation techniques in the absence of median sternotomy or thoracotomy. The first type was a four-step thoracoplasty using titanium plates, implants, and stabilizers, proposed in 2005 by Fette and Rokitansky (27). Furthermore, the use of the Nuss procedure has been reported (28). Finally, Drebov et al. reported a thoracoplasty procedure with fixation using a double-angled mandible locking plate (29).

## Discussion

We present our encouraging but limited experience with this less-invasive device for progressive internal distraction of the sternum in two pediatric patients with congenital JS. There are no guidelines, recommendations, or consensus regarding the ideal surgical approach for JS. Before, during, and after the study period, our institution used only the sternal distraction technique described in this study. None of the other techniques reported in the literature review were used in this study.

Although there is insufficient data to make a reproducible comparison, some theoretical hypotheses can be elaborated. The comparisons were the simplest techniques requiring sternal distraction (group I). In this subgroup, we supported the role of progressive distraction over the prosthesis (subgroup Ib). Even if good results can be obtained with these prostheses, they may be transient. Patients with JS undergo surgery during infancy or the first few years of life, which is a period of rapid growth. Progressive distraction and/or staged procedures may be required to allow progressive chest development to meet a child's overall somatic growth. Moreover, we favor internal devices over external devices regarding progressive distractions. We believe these appear more advantageous and less invasive to the chest.

However, a correct and fair comparison with thoracic expansion techniques (group II) is complex and hazardous because of the completely different access and idea of working. To our knowledge, no comparative studies or series have been conducted in this regard. These approaches have their merits and are described in detail. The invasiveness of a sternotomy should be compared with that of a thoracotomy. Furthermore, sternal spread via median sternotomy vs. thoracotomy-induced thoracic expansion (lateral or vertical) differs completely in terms of the principle and idea behind the dilation increase in the chest volume.

JS is an uncommon syndrome with significant management challenges. Even in specialized centers, surgical care of these patients may be challenging. Only symptomatic patients underwent the procedure at our institution.

Furthermore, the ideal indications and timing for surgery remain unknown because of the lack of guidelines or recommendations. In our experience and opinion, the best timing is when patients begin to exhibit moderate-to-severe symptoms but still have a good chance of experiencing clinical improvement. The respiratory status evolves and progresses with a rapid decline in lung function that usually results in death, frequently in the first few months or years of life (2). Moreover, since patients with mild or intermediate forms at diagnosis frequently develop recurrent respiratory infections, the risk of respiratory deterioration is significant even during follow-up (3).

Evidence for the surgical treatment of JS is extremely scarce owing to its rarity. Various surgical techniques have been proposed for this purpose. These techniques are dissimilar in invasiveness, tools, and access. Most of these techniques have only been proposed and documented in isolated cases because of the extreme rarity of the syndrome and the frequent heroic treatment setting (7–13, 15–17, 20, 24, 27, 29).

To our knowledge, no literature review has focused on different surgical approaches for JS. Furthermore, JS attracts very little public interest compared to other rare diseases or syndromes. For pediatric thoracic surgeons, the lack of guidelines, consensus, or recommendations regarding surgery is a major problem that makes caring for patients with JS even more difficult. Moreover, the lack of evidence makes it impossible to properly inform family members and caregivers about patient outcomes. To contextualize our experience and organize the various existing approaches, we reviewed the contemporary English-language literature on the techniques currently used for surgical treatment in children with congenital JS.

We developed a classification system based on the surgical board and principles of the abovementioned techniques listed in Table 1. Our aim in creating this classification was to reduce the confusion caused by the large number and variety of reported operations.

Our technique is classified in this new classification under subgroup Ib, which includes devices for progressive distraction. An internal distraction device was used for distraction. As there is no specific tool for internal sternal distraction, we used a minimally invasive technique and a small device (Zurich II Micro Modular Distractor, KLS Martin Group, Germany) adapted from maxillofacial surgery. Our approach can be seen as a modification and advancement of the technique first proposed by Conroy et al. in an isolated case (10). Leibinger MIDs (Stryker Leibinger, Germany) have been used to treat craniofacial disorders, and these authors were the first to describe their use for congenital JS. Because no specific devices were available, surgeons frequently developed and/or utilized new custom-made tolls (e.g., modified Bailey Rib Approximator) (9) or devices originally developed for different uses (e.g., the present study) (10). However, the unbalanced sizes of these adapted or modified devices may increase their invasiveness and morbidity.

## Conclusions

Our experience shows promising results regarding the safety and effectiveness of this less-invasive device for PIDS in patients with symptomatic JS. Further studies on long-term outcomes are needed to validate these findings.

## Data Availability

The original contributions presented in the study are included in the article/Supplementary Material, further inquiries can be directed to the corresponding author.
